# Patient Safety Incidents and Nursing Workload**^1^**

**DOI:** 10.1590/1518-8345.1280.2841

**Published:** 2017-04-06

**Authors:** Katya Cuadros Carlesi, Kátia Grillo Padilha, Maria Cecília Toffoletto, Carlos Henriquez-Roldán, Monica Andrea Canales Juan

**Affiliations:** 2 PhD, Adjunct Professor, Facultad de Enfermería, Universidad Andrés Bello, Santiago de Chile, Chile.; 3 PhD, Full Professor, Escola de Enfermagem, Universidade de São Paulo, São Paulo, SP, Brasil.; 4 PhD, Associate Professor, Facultad de Enfermería, Universidad Andrés Bello, Santiago de Chile, Chile.; 5 PhD, Full Professor, Instituto de Estadística, Facultad de Ciencias, Universidad de Valparaíso, Valparaíso, Chile.; 6 MSc, Assistant Professor, Facultad de Enfermería, Universidad Andrés Bello, Santiago de Chile, Chile.

**Keywords:** Patient Safety, Nursing, Nursing Team, Workload, Patient Harm

## Abstract

**Objective:**

to identify the relationship between the workload of the nursing team and the occurrence of patient safety incidents linked to nursing care in a public hospital in Chile.

**Method:**

quantitative, analytical, cross-sectional research through review of medical records. The estimation of workload in Intensive Care Units (ICUs) was performed using the Therapeutic Interventions Scoring System (TISS-28) and for the other services, we used the nurse/patient and nursing assistant/patient ratios. Descriptive univariate and multivariate analysis were performed. For the multivariate analysis we used principal component analysis and Pearson correlation.

**Results:**

879 post-discharge clinical records and the workload of 85 nurses and 157 nursing assistants were analyzed. The overall incident rate was 71.1%. It was found a high positive correlation between variables workload (r = 0.9611 to r = 0.9919) and rate of falls (r = 0.8770). The medication error rates, mechanical containment incidents and self-removal of invasive devices were not correlated with the workload.

**Conclusions:**

the workload was high in all units except the intermediate care unit. Only the rate of falls was associated with the workload.

## Introduction

In the past 15 years, the concern for patient safety has been a priority, motivating proposals of international health policies, and leading to joint efforts of institutions, health professionals and patients in order to reduce and effectively control the risks originated in the health services.

In Latin America, incidents of patient safety, defined as an event or circumstance that may have or effectively have caused unnecessary harm to patients, including incidents related to medication dispensing, falls, accidents with patients, medical equipment and infections associated with health care^1^, happen in 10% of hospitalized patients^2^. In Chile, studies have reported prevalence ranging between 6.2% and 15.7% of incidents^3^ ^-^ ^4^.

The extensive line of research developed worldwide, has identified risk factors associated with patients and health organizations. In the latter are included those related to nursing work environments, such as: leadership, organizational structure of work, academic environment, burnout and workload of the nursing team, among others^5^.

In this area, studies have explored the association between the workload of the nursing team and the quality and safety of care given to the patient, showing an inverse relationship between the nurse/patients ratio and negative effects for patients and nurses. Among the negative effects for patients: increases in the failure to rescue; increased incidence of urinary tract infection; pneumonia and upper gastrointestinal bleeding; patients' falls and increased mortality(6-8). Furthermore, research has shown that insufficient nursing staffing cause job dissatisfaction, stress and intention of quitting the job^9^ ^-^ ^11^.

It is noteworthy that nursing workload is defined as a product of the average daily number of patients seen, adjusted by the degree of dependence and type of care, the average time of assistance for each patient, according to dependence and type of care delivered^12^. The instrument *Therapeutic Intervention Scoring System* (TISS-28) has been the most widely used and recognized worldwide tool for measuring nursing workload in the context of critical patients. The measurement is performed by the procedures performed on the patient and as a result a single TISS-28 point corresponds to 10.6 minutes time of a nurse in direct care^13^.

Despite the relevance at an international level, nursing staffing levels differ widely between hospitals and inpatient units of the same specialty with the same level of dependence of patients^14^ ^-^ ^15^.

This fact, coupled with the need to improve the security of the environment to provide care, has led in some developed countries to the issue of regulations related to nurse/patient ratios.

In 2005, Chile launched a new health reform that included quality assurance for patients. This initiative materialized in public policies and programs for patient safety, among the most outstanding: the law of rights and duties of patients, the regulations for patient safety and the quality evaluation system for institutional providers^16^. However, there are no mandatory standards for baseline staffing numbers for nurses and technical nurses, by different type of units, according to the patient's needs or any other criteria.

In this context, the theme of the impact of staffing levels on patient safety, prompted us to conduct a study covering all inpatient units of a high complexity hospital, as a way to answer the following question: Is there a relationship between the workload of the nursing team and the occurrence of patient safety incidents related to nursing care in a hospital of high complexity of Chile? Thus, the aim of this study was to identify the relationship between the workload of the nursing team and the occurrence of patient safety incidents related to nursing care in a public hospital of high complexity of Chile.

## Methods

It consisted of a cross-sectional analytical study, developed in a public hospital of high complexity of the city of Viña del Mar, Chile, in December 2011, January and February 2012.

The following adult patients hospitalization services were included: cardiovascular intensive care unit, general intensive care unit, intermediate care unit, psychiatry, surgical specialties, medical and surgical public, surgery, medicine, oncology, medical-surgical private patients and pediatric unit.

The population totaled 3430 patients hospitalized in the 11 services considered as strata. From them, it was defined as a stratified and proportional probability sample comprising 879 patients. The population of the nursing team corresponded to all nurses and nursing assistants who were carrying out their functions in the 11 participating services during the study period. In total, there were 85 nurses and 157 nursing assistants.

The response variable selected was the incident of patient safety, which was classified into: errors in dispensing medication, patient falls, self-removal of invasive devices and incidents associated with mechanical containment; and as an explanatory variable: the workload nurses and nursing assistants.

The workload of nurses was defined as the ratio of nurses compared with the number of patients hospitalized; for the medical-surgical and intermediate care units while the Spanish version of the *Therapeutic Intervention Scoring System* (TISS-28) was used for intensive care units. To compare the optimal standard of the number of nurses in medical-surgical units, and given the absence of national standards, it was used the recommendation of the State of California, USA, 2011^17^.

Finally, the workload of nursing assistants was defined as the ratio between the number of nursing assistants compared with the number of patients hospitalized in medical-surgical units, intermediate care and intensive care units.

For the study of patient safety incidents with and without damage, the observation unit was the patient's clinical record. Medical records made throughout the hospitalization of discharged patients were reviewed. To study the nursing workload we used the census of hospital patients, the daily report of the nurses' staffing plan for both day and night shifts and the result of TISS-28. Data were collected on specific instruments and entered in an Excel spreadsheet to be then analyzed in the Data Analysis and Statistical Software (Stata) program. The data were collected by one of the authors, following approval of the ethics committee and institutional management.

Both univariate descriptive and multivariate analyses were performed. Usual descriptive statistics were obtained, as well as specific prevalence rates by type of incident. The overall rate of medication errors corresponded to the ratio between the number of medication errors and the number of error opportunities multiplied by 100. The fall rate was calculated by dividing the number of falls by the number of hospital days multiplied by 1000. The rate for self-removal of invasive devices was calculated by dividing the number of self-removal incidents by the number of hospitalized patients and then multiplying by 100. Finally, the incident rate associated with mechanical restraint was obtained from the ratio between the number of these incidents and the number of hospitalized patients and then multiplying by 100. Pearson correlation was calculated between quantitative variables.

To estalish the associations between the variables studied was used the principals components analysis (PCA), that is a statistical technique of multivariate analysis that aims to reduce the dimensions of analysis, i.e., the number of variables, into a smaller amount showing in a simpler way those that are highly correlated. The new selected variables correspond to linear combinations of the previous, that are being built in order of importance referred to the full variability from the sample. So then, the first principal component will retain variables with highest correlation in absolute terms (positive and negative). The second component holds the second highest degree of association, practically discarding the detected in the first component and so on. Usually, the first two or three components explain the greater association between the variables studied.

The study unit was defined as each one of the 11 clinical services. For this end, 16 variables were analyzed: 12 of them were explanatory, corresponding to the workload for the day and night shifts (12 hours each), for nurses and nursing assistants, per month; and four response variables corresponding to the specific incident rates: medication errors, falls, self removal of invasive devices and incidents associated with mechanical restraint.

This research was approved by the Ethics Committee of the Naval Hospital by Resolution PO10/09, according to the institutional and national legislation for approval of research involving human subjects.

## Results

In the study were detected a total of 625 patient safety incidents with an average of 0.7 incidents per patient. Medication errors accounted for 89.56% (n = 558) of all incidents, followed by self-removal of invasive devices, 5.29% (n = 33), incidents related to containment 3.52% (n = 22) and finally falls representing 1.92% (n = 12).

The overall incident rate was 71.1%. The intermediate care unit had the highest rate with 129.8%, closely followed by the medicine ward with 128.8%. The lowest rates were recorded in oncology with 0%, followed by pediatric and medical-surgical private patients with 12.4% and 12.9% respectively (Table 1).

**Table 1:** Distribution of patient safety incidents by type and clinical service. Viña del Mar, Chile, 2011-2012

Service	Medication Error	Patient Safety Incidents				
		Self removal of invasive	Falls	Mechanical Restraints Incidents	Total	Global Rate (%)
Medical-Surgical Public	22	4	2	2	30	28,6
Medicine	234	15	6	9	264	128,8
Surgical Specialties	44	5	1	4	54	56,2
Oncology	0	0	0	0	0	0
Surgery	136	5	3	5	149	75,2
Pediatrics	11	0	0	0	11	12,4
Intermediate Care Unit	58	2	0	1	61	129,8
Intensive Care UnitCardiovascular	16	1	0	1	18	37,5
Cardiovascular Intensive Care Unit	17	1	0	0	18	51,4
Psychiatry	11	0	0	0	11	42,3
Medical-Surgical Private	9	0	0	0	9	12,9
Total	558	33	12	22	625	71,1

The overall medication error rate obtained for the study sample was 0.9%. The highest rate was recorded in the pediatric ward with 1.5%, followed by medicine with 1.4% and the lowest in oncology with 0%.

The overall fall rate was 2.0 per 1,000 days of hospitalization. The service with higher rates of falls was medicine with 3.6 per 1,000 days of hospitalization, followed by medical and surgical institutional with 3.2 per 1,000 days of hospitalization. Zero rate was recorded in oncology, pediatric, medical-surgical private patients, psychiatry, general intensive care unit, cardiovascular intensive care unit and intermediate care unit services.

The overall rate of self-removal of invasive devices was 5.5 per 1,000 days of hospitalization. The service with the highest rate was medicine with 8.9 per 1,000 hospital days, followed by surgical specialties with 8.4 per 1,000 days of hospitalization. Zero rate was recorded in oncology services, pediatrics, medical-surgical private patients and psychiatry.

Finally, the overall rate of incidents associated with mechanical restraint was 2.5 per 1,000 days of hospitalization. The service with highest rate was surgical specialties, with 6.7 per 1,000 days of hospitalization, followed by cardiovascular intensive care unit with 6.4 per 1000 days of hospitalization. Zero rate corresponded to oncology services, pediatrics, medical-surgical private patients, psychiatry and general intensive care unit.

Regarding the workload in the medical-surgical and specialty services, the highest workloads for nurses and nursing assistants per patient were observed in the services of medicine, surgery and surgical specialties (for nurses varied 1:20.5 to 1:24.5 patients on day shift and 1:48 to 1:57.3 in night shift, and for nursing assistants ranged from 1:6.2 to 1:7.6 patients on the day shift and 1:7.2 to 1:9.7 patients in night shift). Pediatrics was the service with less workload (for nurses ranged from 1:4.8 to 1:5.0 patients on day shift and 1:6.4 to 1:7.0 patients on nightshift and, for nursing assistants it ranged from 1:1.9 to 1:2.9 patients on the day shift and 1:2.5 to 1:3.8 patients on the night shift).

The TISS-28 showed more marked variations in general intensive care unit. While in the cardiovascular intensive care unit the TISS average ranged between 91.2 and 116.8, in the general intensive care unit ranged between 108.9 and 206.6. The general intensive care unit had a greater number of days understaffed (n = 79) while in the cardiovascular intensive unit, the understaffed days were 55. The days with adequate staffing according to TISS were only 10 in the general intensive care unit and 28 in the cardiovascular intensive care. Additionally, the days according to the TISS with extra staffing were 8 in the cardiovascular intensive care and only 2 in general intensive care unit (Table 2).

**Table 2:** Average staffing required as per TISS-28, minimum, maximum and adequate by month in Intensive care Units. Viña del Mar, Chile, 2011-2012

Service	Month	TISS	AST*	Min AST^†^	Max AST^†^	Days A^‡^	%	Days I^§^	%	Days E^||^	%
Cardio vascular Unit	Dec	116,8	2,5	0,7	2,5	3	9,7	26	83,9	2	6,4
	Jan	95,5	2,0	0,3	3,5	10	32,2	16	51,6	5	16,1
	Feb	91,2	1,9	1,0	3,4	15	51,7	13	44,8	1	3,4
General Unit	Dec	206,6	4,4	2,6	6,3	0	0	31	100,0	0	0
	Jan	138,9	3,0	1,6	5,0	3	9,7	28	90,3	0	0
	Feb	108,9	2,3	0,9	3,2	7	24,1	20	68,9	2	6,9

* Average Staffing according to TISS-28; ^†^ Required staffing according to TISS-28; ^‡^ Adequate Nursing staffing according to TISS-28; ^§^Insuficient Nursing staffing according to TISS-28 ^||^ Extra Nursing staffing according to TISS-28

In an analysis of the data from a multivariate perspective, the results for the first three principal components explained 95.8% of the variability of the set of 16 variables. The first component explained 80.9% of the total variability; while the second component, explained 9.1% and, finally, the third component explained 5.8% of the total variability.

The association between the 16 variables -12 of them were workload variables plus four incidents variables, and the first three principal components, resulted in the following: for the first component there was a very high positive correlation between all the variables of workload with a correlation that ranged between 0.9611 and 0.9919. In turn, a very high positive correlation between workload variables and the rate of falls (r = 0.8770) was recorded. For the second main component, there was correlation between the rate of self-removal of invasive devices (r = 0.7744) and the incident rate associated with mechanical restraint (r = 0.7748). However, no correlation was evident between these variables and the workload. Finally, in the third principal component, only the variable medication error rate presented a high correlation index (r = 0.9124) but no correlation was found with the other studied variables (Table 3).

**Table 3:** Association between the 16 variables with the three principal components. Viña del Mar, Chile, 2011-2012

Variable	Component 1	Component 2	Component 3
Rate of self removal of invasive	0,5505	0,7744	-0,2171
Rate of falls	0,877	0,1606	-0,0105
Rate associated with mechanical restraint	0,541	0,7748	-0,1167
Rate of medication error	0,28	0,2941	0,9124
Workload auxiliaries December day	0,9599	0,0392	-0,0932
Workload auxiliaries January day	0,9919	-0,0713	0,0089
Workload auxiliaries February day	0,9804	-0,1238	0,0315
Workload Nurses December day	0,9935	0,0115	0,0075
Workload nurses January day	0,9741	-0,1458	0,0286
Workload nurses February day	0,9892	-0,1352	-0,0064
Workload auxiliaries December night	0,9657	-0,0793	-0,1295
Workload auxiliaries January night	0,9797	-0,1383	-0,0066
Workload auxiliaries February night	0,9611	-0,1719	0,0056
Workload nurses December night	0,989	-0,0587	0,0432
Workload nurses January night	0,9813	-0,0972	0,0269
Workload nurses February night	0,9805	-0,1213	0,0144

## Discussion

The workload of nurses, as measured in this study, was found to be over the international recommendations and over those standards of TISS-28, except for intermediate care unit on day and night shift and pediatrics at day shift .

Medicine, surgery and surgical specialties were the services with the largest number of patients per nurse (one nurse: 20.5 to 24.5 patients on day shift and one nurse: 48 to 57.3 in night shift). The higher proportion of patients to be cared of, was recorded at night shifts and weekends. The workload of nursing assistants was also higher in medicine, surgery and surgical specialties. In these services the ratio nursing assistants/patients ranged from an auxiliary to 6.0 to 7.6 patients on the day shift and an auxiliary to 8.2 to 9.7 patients on the night shift.

These results indicate a higher ratio than stated in some international studies^6^ ^,^ ^18^; however, we must consider that the workload estimation used in this study (exception made for the intensive care units) did not consider risk or degree of dependence of patients, a condition that should be taken into account when making comparisons.

The only patient safety incident that showed high correlation with the independent variables analyzed was the fall rate of patients. The high positive correlation observed between the workload of the services studied and the rate of falls, shows a scenario in which the amount of activities to be developed in a large number of patients probably exceeded the ability to respond to the care needs and patient monitoring, and this may explain the frequence of falls. Similar results were obtained by other authors, whose studies also correlated the high workload with inpatient falls^6^ ^,^ ^19^.

Oncology was the only service that did not recorded incidents of any kind; however, the workload was high in this service. The literature describes prevalence of falls between 15% and 17%^20^ and medication error rate of 13.6% for these units^21^.

Psychiatry did not record falls, incidents associated with self-removal of invasive devices or incidents associated with mechanical restraint, despite having a high workload. Literature describes fall rates at 1.5 falls per 1000 days of hospitalization in psychiatric patients^22^ and the occurrence of adverse events associated with mechanical restraint that can range from simple injuries to death^23^. Thus, the results obtained by these services could be explained by non-studied variables, or by the complex interplay between organizational variables that can determine different points of balance or compensation acting as protective factors in a particular clinical service. However, this also needs to be further explored.

Medication errors were not correlated with any of the explanatory variables, although others studies have described the relationship between them and staffing levels(6,24), suggesting that its occurrence might also be influenced by factors not studied in this investigation. Some of these could be active failures, degree of adherence to protocols and monitoring, among others. These variables can be studied in further investigations in order to elucidate the factors associated with them.

The complex scenario for clinical safety involving simultaneous care of a large number of patients, according to what was observed in this research, raises the need for care models that include determining the nurse time-needs per patient, considering the demands care, stage of life cycle, seasonal contingencies and complexity of nursing activities. All these parameters have variations by type of service and patient. This is a challenge for health institutions, in their ability to adapt to the changing needs of the environment, through human resources management strategies that are at the same time safe, innovative, efficient and patient-centered.

For the Chilean reality these are gaps that still remain to be solved, and more studies are necessary to make a diagnosis at country level. This is a necessary condition for designing new models and health policies related to people management of the nursing team focused on patient safety.

Regarding limitations, the observation units for the study were clinical services, leading to a reduction of the universe of the study to 11 units, which precluded traditional multivariate techniques (multiple regression and multiple logistic regression).

The variety of methods used by the studies, as well as the variables and formulas used to calculate incidence and prevalence of patient safety incidents made difficult to compare results with other scientific publications. Finally, this study was conducted in only one hospital and, given the characteristics of the type of statistical analysis used, the results are valid only for this hospital.

## Conclusions

The workload observed in this study, as expressed by the ratio of number of patients to be attended per nurse and nursing assistants, was higher in all units except the intermediate care unit. Medicine, surgery and surgical specialties were the services with the highest workload.

Patients' falls were the only incident associated with the workload of nurses and nursing assistants. Contrary to evidence, medication errors, self removal of invasive and adverse events associated with mechanical restraint showed no association with any of the workload variables studied.

The results realized the complexity of the environment in which nursing care can be delivered, leaving open for new questions of research, regarding the identification of variables that could explain the occurrence of the incidents for which no association was found with workload.

Moreover, these results reveal the need to deepen the knowledge regarding the estimation of staffing needs considering the particular characteristics of the epidemiological reality and those specific to the nursing care as practised in Chile.
